# Leafhopper-Induced Activation of the Jasmonic Acid Response Benefits *Salmonella enterica* in a Flagellum-Dependent Manner

**DOI:** 10.3389/fmicb.2018.01987

**Published:** 2018-08-23

**Authors:** Kimberly N. Cowles, Russell L. Groves, Jeri D. Barak

**Affiliations:** ^1^Department of Plant Pathology, University of Wisconsin–Madison, Madison, WI, United States; ^2^Department of Entomology, University of Wisconsin–Madison, Madison, WI, United States

**Keywords:** biomultiplier, jasmonic acid, bacterial flagellum, plant immunity, pathogen-triggered immunity

## Abstract

Enteric human pathogens such as *Salmonella enterica* are typically studied in the context of their animal hosts, but it has become apparent that these bacteria spend a significant portion of their life cycle on plants. *S. enterica* survives the numerous stresses common to a plant niche, including defense responses, water and nutrient limitation, and exposure to UV irradiation leading to an increased potential for human disease. In fact, *S. enterica* is estimated to cause over one million cases of foodborne illness each year in the United States with 20% of those cases resulting from consumption of contaminated produce. Although *S. enterica* successfully persists in the plant environment, phytobacterial infection by *Pectobacterium carotovorum* or *Xanthomonas* spp. increases *S. enterica* survival and infrequently leads to growth on infected plants. The co-association of phytophagous insects, such as the Aster leafhopper, *Macrosteles quadrilineatus*, results in *S. enterica* populations that persist at higher levels for longer periods of time when compared to plants treated with *S. enterica* alone. We hypothesized that leafhoppers increase *S. enterica* persistence by altering the plant defense response to the benefit of the bacteria. Leafhopper infestation activated the jasmonic acid (JA) defense response while *S. enterica* colonization triggered the salicylic acid (SA) response. In tomato plants co-treated with *S. enterica* and leafhoppers, both JA- and SA-inducible genes were activated, suggesting that the presence of leafhoppers may affect the crosstalk that occurs between the two immune response pathways. To rule out the possibility that leafhoppers provide additional benefits to *S. enterica*, plants were treated with a chemical JA analog to activate the immune response in the absence of leafhoppers. Although bacterial populations continue to decline over time, analog treatment significantly increased bacterial persistence on the leaf surface. Bacterial mutant analysis determined that the bacterial flagellum, whether functional or not, was required for increased *S. enterica* survival after analog treatment. By investigating the interaction between this human pathogen, a common phytophagous insect, and their plant host, we hope to elucidate the mechanisms promoting *S. enterica* survival on plants and provide information to be used in the development of new food safety intervention strategies.

## Introduction

According to the Centers for Disease Control and Prevention (CDC), the human enteric bacterial pathogen *Salmonella enterica* is estimated to cause disease in more than 1.2 million people annually in the United States. Salmonellosis is most often characterized by gastroenteritis that may result in diarrhea, fever, and abdominal cramping. Of the *S. enterica*-related illnesses that are acquired in the United States, more than 90% are estimated to have come from a food source with 46% involving fresh produce. *S. enterica* is ubiquitous in the plant environment, is commonly isolated from water sources used for irrigation or pesticide application, and persists for months on the roots and leaves of agricultural crops (Islam et al., 2004a,b; Haley et al., 2009). The phyllosphere (the above-ground parts of the plant) is a hostile environment with fluctuations in temperature, water and nutrient availability, and UV irradiation (O’Brien and Lindow, 1989; Islam et al., 2004b; Barak and Liang, 2008; Poza-Carrion et al., 2013). The ability of *S. enterica* to overcome these environmental stresses contributes to the incidence of human disease due to the consumption of contaminated fresh produce (Gorski et al., 2011; Painter et al., 2013).

To survive on the leaf surface, epiphytic bacteria actively migrate to preferential niches such as trichomes, stomates, and cell junctions (Haefele and Lindow, 1987; Kinkel, 1997; Monier and Lindow, 2003; Remus-Emsermann et al., 2012). In these sites, bacteria find increased nutrient availability and protection against fluctuations in temperature, humidity, and UV radiation (Leveau and Lindow, 2001). In general, bacteria utilize many different types of motility, including swimming, swarming, twitching, gliding, and sliding (Henrichsen, 1972; Macnab, 1996; Mattick, 2002; Harshey, 2003; Mignot et al., 2007; Kearns, 2010). Perhaps the most well studied, swimming motility utilizes the flagellum to propel a bacterium through a semi-solid or liquid medium. The flagellum is a complex organelle in terms of both structure and regulation and is important for bacterial survival in many niches, including the surfaces of leaves (for recent review, see Rossez et al., 2015). For example, the plant pathogen *Pseudomonas syringae* requires flagellum-dependent swimming motility for full fitness on the surface of bean leaves. Motility mutants are less resistant to UV irradiation and desiccation, and mutant populations are reduced compared to the wildtype parent in colonization assays (Haefele and Lindow, 1987; Lindow et al., 1993). Similarly, *S. enterica* utilizes flagella for swimming and swarming motility, and flagella contribute to bacterial attachment to basil leaves (Berger et al., 2009).

Although the bacteria successfully locate preferred sites on the leaf surface, *S. enterica* populations slowly decline over time (Islam et al., 2004a,b; Barak et al., 2011). Unlike phytobacterial pathogens, *S. enterica* cannot liberate nutrients from the plant, leaving the human pathogen in an environment where resources are limiting. However, biotic factors, such as infection with phytobacterial pathogens, can release nutrients and allow for proliferation or enhanced survival of human bacterial pathogens. Depending on the species, *Xanthomonas*-induced necrosis and water-soaking leads to increased persistence of *S. enterica* serovar Typhimurium (*S.* Typhimurium), or even replication in the case of *Xanthomonas euvesicatoria* and *Xanthomonas gardneri*, on tomato leaves (Potnis et al., 2014, 2015). Similarly, tissue damage caused by the soft-rotting bacterium *Pectobacterium carotovorum* subsp. *carotovorum* enhances *S.* Typhimurium persistence on romaine lettuce leaves (Kwan et al., 2013). Recently, our lab expanded the list of biological factors that enhance *S. enterica* survival (biomultipliers) to include phytophagous insects and demonstrated that insect infestation increases *S. enterica* persistence on plants (Soto-Arias et al., 2013, 2014). Specifically, the presence of Aster leafhopper (*Macrosteles quadrilineatus*), a common phloem-feeding pest of agricultural crops, significantly improved *S. enterica* persistence on lettuce leaves (Soto-Arias et al., 2013). Furthermore, phytophagous insects that encounter *S. enterica*-contaminated plants represent a risk for transmitting disease as they frequently move within and between plants while feeding and can be contaminated both externally and internally with *S. enterica* (Soto-Arias et al., 2014). Thus, phytophagous insects represent a risk factor for the dispersal of *S. enterica* amongst plants in an agricultural setting.

The mechanism(s) by which leafhopper infestation promotes *S. enterica* persistence on plants are unknown. Reviewing the list of environmental stresses present in this environment, we hypothesize that leafhoppers alter the plant immune response in a way that benefits bacterial survival on leaves. Plant immunity is characterized by a multi-layer defense response that is reminiscent of animal innate immunity. Pattern recognition receptors (PRRs) detect conserved pathogen associated molecular patterns (PAMPs) from invading microorganisms which trigger a cascade of responses (for reviews, see Garcia and Hirt, 2014; Melotto et al., 2014). Initially, the plant responds within seconds to minutes with changes in ion fluxes and extracellular alkalinization and increased production of reactive oxygen species (ROS). These changes are followed in the next minutes to hours with increases in ethylene production, stomatal closure, mitogen-activated protein kinase (MAPK) signaling, and transcriptional reprogramming. Within hours to days, accumulations in callose [β-(1,3)-glucan polymer] deposition, salicylic acid (SA) production, and defense gene transcription are observed. The defense hormones SA and jasmonic acid (JA) are well characterized signaling hormones critical to effective plant immunity. Generally, the SA pathway is induced by the plant in response to biotrophic pathogens while the JA pathway is activated in the presence of necrotrophic pathogens or chewing herbivores. Although not a strict rule, the SA and JA pathways are typically antagonistic; activation of one pathway leads to inhibition of the other (Pena-Cortes et al., 1993; van Wees et al., 2000; Spoel, 2003; Van der Does et al., 2013; Wei et al., 2014). Crosstalk between the two pathways is thought to fine tune the plant immune response to pathogens (Beckers and Spoel, 2006; Koornneef and Pieterse, 2008; Pieterse et al., 2012). Disruption or alteration of the SA and JA pathways could have downstream effects on the plant immune response and impact microbial survival.

Aster leafhoppers feed by inserting their stylets through plant cells to reach the phloem. Although this feeding style causes little cellular damage compared to chewing herbivores such as caterpillars, leafhoppers induce the JA response in *Arabidopsis thaliana* and tobacco (*Nicotiana attenuata*) plants (Sugio et al., 2011; Kallenbach et al., 2012). In contrast, in *A. thaliana*, *Medicago truncatula*, and lettuce (*Lactuca sativa*), inoculation with *S. enterica* leads to induction of the SA pathway (Iniguez et al., 2005; Klerks et al., 2007; Schikora et al., 2008; Jayaraman et al., 2014). Phytophagous insects have developed strategies to manipulate plant immunity to the benefit of the insect. As one example, the Colorado potato beetle (*Leptinotarsa decemlineata*) utilizes bacteria found in the beetle’s oral secretions to reduce the JA response to the beetle by preemptively activating the SA response using the oral bacteria (Chung et al., 2013a,b). In this way, the beetle manipulates the host into responding to the bacteria, taking advantage of the antagonistic nature of the JA and SA pathways, and giving the herbivore, and their offspring, easier access to plant nutrients. The interaction between *S. enterica* and leafhoppers may not be as directly symbiotic as the Colorado potato beetle and its oral microflora, but we hypothesize that leafhopper-induced activation of the JA response may lead to antagonistic suppression of the SA response and indirectly create a more permissive environment for *S. enterica*.

In this study, we tested the hypothesis that infestation with leafhoppers alters the plant immune response and increases *S. enterica* persistence. Several multistate and international outbreaks have been linked to *Salmonella*-contaminated tomatoes (Cummings et al., 2001; Centers for Disease Control and Prevention, 2005, 2007; Gupta et al., 2007; Greene et al., 2008), making tomato a relevant plant host for studying *S. enterica* persistence. We chose to examine tomato plants as a way to compare the effects of leafhoppers on *S. enterica* populations in a second plant host. Additionally, we used the JA-inducible proteinase inhibitor gene *pin1* and the SA-inducible pathogenesis related protein gene *pr1a1* as established markers (Tornero et al., 1994; Fowler et al., 2009) to monitor the two plant defense responses with quantitative PCR. Further, we examined the effect of a chemical JA analog on *S. enterica* persistence and investigated the importance of the bacterial flagellum in this system.

## Materials and Methods

### Bacterial Strains, Media, and Culture Conditions

The nalidixic acid-resistant strain of *S. enterica* serovar Typhimurium 14028s (*S.* Typhimurium; Cowles et al., 2016) was used as wildtype in this study (**Table 1**). The *fliF*::Kan mutant was generated using primers listed in **Table 2** with the λRed recombinase method (Datsenko and Wanner, 2000), and the *motA::*Kan mutant was obtained from a mutant library collection (Santiviago et al., 2009). These deletion–insertion mutations were transduced into a fresh *S.* Typhimurium background using P22 (Gemski and Stocker, 1967) and confirmed by PCR (see **Table 2** for confirmation primers). Bacterial cultures were grown in lysogeny broth (LB) at 37°C with shaking at 200 rpm. The antibiotics nalidixic acid (Nal) and kanamycin (Kan) were used at concentrations of 20 and 50 μg/ml, respectively. Strains used in this study are shown in **Table 1**, and primers used to confirm bacterial mutants are shown in **Table 2**.

**Table 1:** List of strains.

Strain designation	Genotype	Reference or source
***Salmonella enterica* strains**		
JDB682	*S. enterica* serovar Typhimurium 14028s	ATCC
JDB1034	*S. enterica* serovar Typhimurium 14028s; Nal^R^	This study
JDB903	Δ*fliF*::Kan	This study
JDB902	Δ*motA*::Kan	This study

**Table 2:** List of primers.

Primer	Primer sequence (5′–3′)		% Efficiency
	Forward	Reverse	
**Quantitative PCR analysis^a^**			
*act41*	GCTCTTGACTATGAACAGGAAC	AAGGACCTCAGGACACCG	104
*ubi3*	GCCGACTACAACATCCAGAAGG	TGCAACACAGCGAGCTTAACC	105
*pin1*	GCTAAGGAAATAATTGAGAAGGA	TAAGTCACCACAGGCATT	102
*pr1a1*	TCAAAGAGCTGATGACTGTG	GTACCATTGCTTCTCATCGT	102
**Mutant construction and confirmation^b^**			
*fliF*::Kan construction	ATGAGTGCGACTGCATCGACTGC AACCCAACCTAAACCTCGTGTA GGCTGGAGCTCCTTC	GATCGTTACTCATCCACTGGCG AATGACCAGCGCCACC ACCATATGAATATCCTCCTTAG	
*fliF*::Kan confirm	CGTTGCGATGGTGCTGTGGG	GCGCGTCAACTGCGGACGTA	
*motA*::Kan confirm	GCGGACACCTTGGGGCACTC	TGGACGCTCACTGGAATAAAGCG	

*^a^Quantitative PCR primers were used to examine transcription of plant genes (as described in the Section “Materials and Methods”). ^b^Mutant construction primers were used for the λRed recombinase method, and mutant confirmation primers were used to amplify across potential deletion–insertion sites and compare the resulting product sizes to that observed when using wildtype genomic DNA as a template.*

### Insect Rearing

A colony of Aster leafhoppers (*M. quadrilineatus*) was maintained on oat plants (*Avena sativa* L.) in a controlled environment with a 16 h photoperiod with 24°C light and 19°C dark. Even-aged, 3- to 5-day-old adult insects were used for all experiments.

### Plant Inoculation and Infestation

**FIGURE 1:**
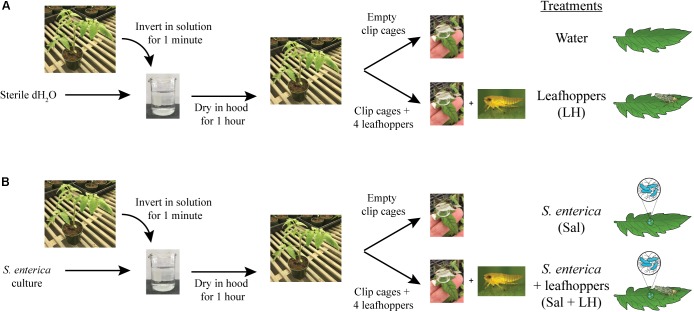
Inoculation and infestation assay. Tomato plants were dip-inoculated by inverting plants in either sterile water **(A)** or bacterial inoculum **(B)**. Dip-inoculated plants were dried for 1 h. Then, half of the water-dipped **(A)** and half of the bacterial-dipped plants **(B)** were fitted with clip cages, to trap insects on the leaflet, containing four adult leafhoppers each. The remaining dip-inoculated plants were fitted with empty clip cages.

*Solanum lycopersicum* (tomato) cultivar MoneyMaker seedlings were cultivated in Professional Growing Mix (Sunshine Redi-earth) with a 16 h photoperiod at 24°C for 5 weeks. For colonization assays, bacterial cultures were grown overnight in LB and normalized to an optical density at 600 nm (OD_600_) of 0.2 in sterile water. An OD_600_ of 0.2 corresponds to a bacterial population level of ∼10^8^ CFU/ml, and this inoculum level was used for all experiments. Prior to inoculation, 0.025% Hi-Wett (Loveland Products, Inc.) was added to water or the bacterial inoculum. Pots containing tomato plants were dip-inoculated by inverting plants in either sterile water or the bacterial inoculum for 1 min with agitation to prevent bacterial cell settlement (**Figure 1**). Dip-inoculated plants were incubated under the blower in a SterilGARD Class II Biosafety Cabinet (The Baker Company) for 1 h to dry the leaves. After the 1-h drying period, half of the water-dipped and half of the bacterial-dipped plants were fitted with empty clip cages on two middle leaflets (as defined in Potnis et al., 2014; **Figure 1**). Clip cages are two cm diameter plexiglass cylinders topped with plastic screen that were attached to the upper leaf surface to trap insects on the leaflet (**Figure 1**). The remaining dip-inoculated plants were fitted with clip cages containing four adult leafhoppers each. Plants were incubated at high humidity in lidded, plastic bins under grow lights with a 16 h photoperiod at room temperature (∼26°C). At multiple time points post-inoculation and/or post-infestation, leaf samples were taken from within clip cages using destructive sampling to determine bacterial populations and collect samples for RNA extraction.

### Chemical Analog Treatment

For exogenous induction of the JA response, 5-week-old MoneyMaker tomato seedlings were sprayed with a fine mist of sterile water or methyl jasmonate (MeJA; 0.000001%; Sigma Aldrich, St. Louis, MO, United States). The MeJA concentration was chosen to most closely mimic the plant response to a leafhopper infestation; optimization experiments determined that there were no observable changes in plant morphology, and the level of JA induction was similar to that seen in response to the insects (data not shown). Plants were incubated for 24 h at high humidity and then dip-inoculated in water or bacterial suspension as described above. Dipped plants were incubated at high humidity as described above. At days 0, 3, 6, 10, and 14 (post-dip inoculation), leaf samples were taken from middle leaflets using destructive sampling to determine bacterial populations and collect samples for RNA extraction.

### Bacterial Population Sampling

At indicated time points, one 79 mm^2^ leaf disc was taken from each of two leaflets on middle leaves (Potnis et al., 2014). Samples from four plants per treatment per time point were transferred to microfuge tubes and homogenized in 500 μl of sterile water using a 4.8 V rotary tool (Dremel, Mount Prospect, IL, United States) with microcentrifuge tube sample pestle attachment (Thermo Fisher Scientific). Homogenates were diluted 1:10 in sterile water and spiral plated (Autoplate 4000, Spiral Biotech, Norwood, MA, United States) on LB Nal plates. Resulting colonies were counted after overnight incubation at 37°C to determine bacterial populations. Experiments were performed with three biological replicates.

### RNA Isolation

At indicated time points, two 79 mm^2^ leaf discs were taken from each of two leaflets on middle leaves (Potnis et al., 2014) and combined for a total of four leaf discs per plant. Samples from four plants per treatment per time point were collected and frozen at −80°C for further processing. RNA was extracted using the PureLink RNA Purification kit (Invitrogen) with some modifications. Briefly, four leaf discs were homogenized with a mortar and pestle in the presence of liquid nitrogen. Ground tissue was transferred to a small weigh boat containing 1 ml Trizol, mixed with a pipette tip, and transferred to a microcentrifuge tube. Samples were then processed according to manufacturer’s instructions and eluted in 100 μl volume of RNase-free water. RNA was treated with Turbo DNA-free (Ambion) for 30 min using the manufacturer’s protocol and quantified by NanoDrop (Thermo Fisher Scientific). RNA was isolated from three biological replicates for each experiment.

### cDNA Synthesis and Real-Time PCR

cDNA synthesis was performed using the iScript cDNA synthesis kit according to manufacturer’s instructions (Bio-Rad) with 1.5 μg total RNA as input. Real-time PCR primers were designed with Beacon Designer software (Premier Biosoft International) avoiding template secondary structure (**Table 2**). Primer efficiencies (**Table 2**) were calculated using serial dilutions of MoneyMaker genomic DNA and CFX Manager 3.0 software (Bio-Rad). Reference transcripts were chosen based on published works: *act41* and *ubi3* (Rotenberg et al., 2006). Stable expression between treatments was validated using the Best Keeper program and four independent RNA samples from each treatment. Real-time PCR experiments were performed as described (Jahn et al., 2008; Cowles et al., 2016). Experiments were performed using the CFX96 Real-Time System and analyzed with the CFX Manager 3.0 software (Bio-Rad). The mean *Cq* of each target transcript was normalized by the mean *Cq* of each reference gene using the formula: 2^(−(*Cq* target − Cq reference))^. As previously described (Rotenberg et al., 2006), we determined the relative expression ratio (RER) of the target gene by dividing the normalized target RNA by a calibrator consisting of the average of the normalized values of the control samples (expression after water treatment in these experiments).

### Statistical Analysis

All statistical analyses were performed using R software (version 2.14.1; R Development Core Team, R Foundation for Statistical Computing, Vienna, Austria^1^) as described (Kwan et al., 2015). Briefly, three biological replicates were performed for each experiment, and samples taken from one replicate were considered as subsamples. To determine whether bacterial population results differed between treatments in the leafhopper and chemical analog experiments, analysis of covariance (ANCOVA) was used as previously described (Soto-Arias et al., 2013), with treatment and time as covariates. For real-time PCR analysis, four samples were compared for each treatment at each time point using Tukey’s HSD test. Results were considered statistically significant at *P* < 0.05.

## Results

### The Presence of Leafhoppers Enhances *S.* Typhimurium Persistence on Tomato Leaves

**FIGURE 2:**
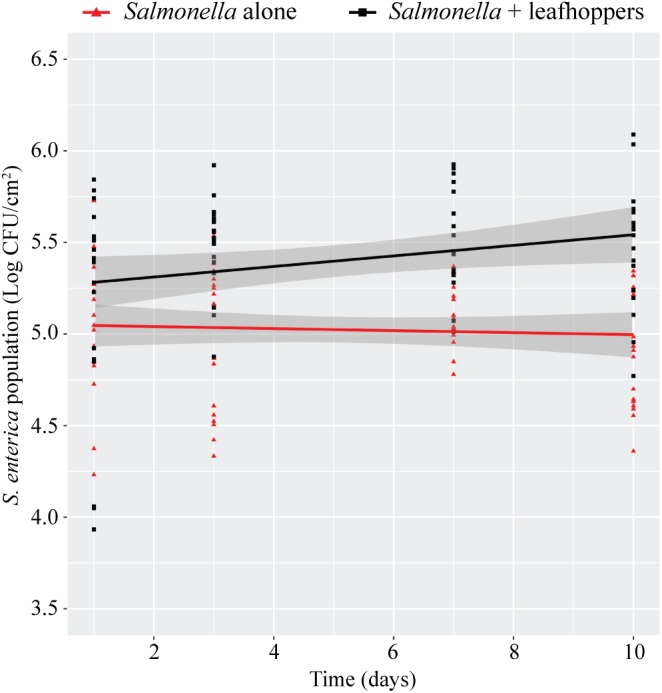
Leafhopper infestation leads to increased persistence of *S.* Typhimurium populations. Bacterial populations on leaves were monitored over time either in the presence (black squares) or absence (red triangles) of leafhoppers. All data points from three independent experiments were combined and presented as *S.* Typhimurium populations (log CFU/cm^2^) over a 10-day period. Lines (black, *S.* Typhimurium with leafhoppers; red, *S.* Typhimurium alone) correspond to a linear regression model, and shaded areas correspond to the respective 95% confidence interval.

Previously, we had shown that infestation with phytophagous insects promotes *S. enterica* survival on the leaves of lettuce plants (Soto-Arias et al., 2013). To determine if this effect occurs in additional plant species, bacterial populations were monitored on tomato plants (*S. lycopersicum*) infested with leafhoppers (**Figure 2**). The presence of leafhoppers significantly enhanced the persistence of *S.* Typhimurium populations over time compared to plants that were inoculated with bacteria alone (**Figure 2**). After 10 days, *S.* Typhimurium populations were approximately ½ log higher in the presence of leafhoppers than in plants that were not infested with insects (**Figure 2**).

### Co-inoculation of *S.* Typhimurium and Leafhoppers Alters the Plant Immune Response

**FIGURE 3:**
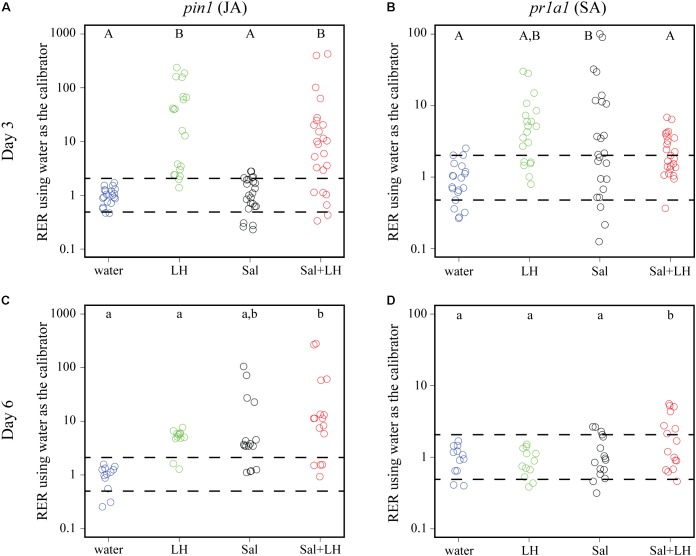
Transcription of plant defense genes changes in response to leafhoppers (LH; green circles), *S.* Typhimurium bacteria (Sal; black circles), or co-inoculation of bacteria with leafhoppers (Sal+LH; red circles). Plant gene expression was quantified at days 3 **(A,B)** and 6 **(C,D)** post-inoculation. Data for the JA-inducible *pin1* **(A,C)** and SA-inducible *pr1a1* **(B,D)** are displayed as relative expression ratios (RER) using transcription in plants treated with water (blue circles) as the negative control and calibrator. The dashed line indicates a twofold change in transcription, and each circle represents data collected from one tomato plant. Transcript levels were quantified from three biological replicates with samples taken from at least four plants per treatment per time point. Letters denote significant differences between treatments within a single time point for that specific plant defense gene (*P* < 0.05).

To test the hypothesis that insect infestation changes the plant immune response to the benefit of *S.* Typhimurium, plant defense gene expression was monitored over time in tomatoes inoculated with leafhoppers, *S.* Typhimurium, or a combination of leafhoppers and *S.* Typhimurium. The JA-inducible proteinase inhibitor gene *pin1* and the SA-inducible pathogenesis related protein gene *pr1a1* were used as established markers (Tornero et al., 1994; Fowler et al., 2009) to monitor the two plant defense responses with quantitative PCR. Day 3 post-inoculation was chosen as the initial time point to monitor plant immune gene expression as it was the time when bacterial populations began to diverge when comparing treatments with or without leafhopper infestation (**Figure 2**). Compared to the negative control (plants treated with water), tomatoes that were infested with leafhoppers showed a significant induction of *pin1* expression and displayed an intermediate level of *pr1a1* expression on day 3 post-inoculation (**Figures 3A,B**). Contrastingly, tomato plants treated with *S.* Typhimurium had no change in *pin1* expression compared to the water control, but had a significant induction of *pr1a1* expression on day 3 (**Figures 3A,B**). Plants that were simultaneously treated with both leafhoppers and *S.* Typhimurium responded with an increase in *pin1* expression but no significant change in *pr1a1* gene expression on day 3 (**Figures 3A,B**). To examine the impacts on later stages of colonization, plant defense gene expression was also measured at day 6 post-inoculation. By day 6, plants that were treated with both leafhoppers and *S.* Typhimurium induced both *pin1* and *pr1a1* while treatment with *S.* Typhimurium alone led to an intermediate induction of *pin1* expression compared to the water control (**Figures 3C,D**). By day 6, *pin1* and *pr1a1* levels were not significantly different from the negative control following leafhopper infestation (**Figures 3C,D**).

### Exogenous Treatment With Methyl Jasmonate Is Sufficient to Increase Bacterial Persistence

**FIGURE 4:**
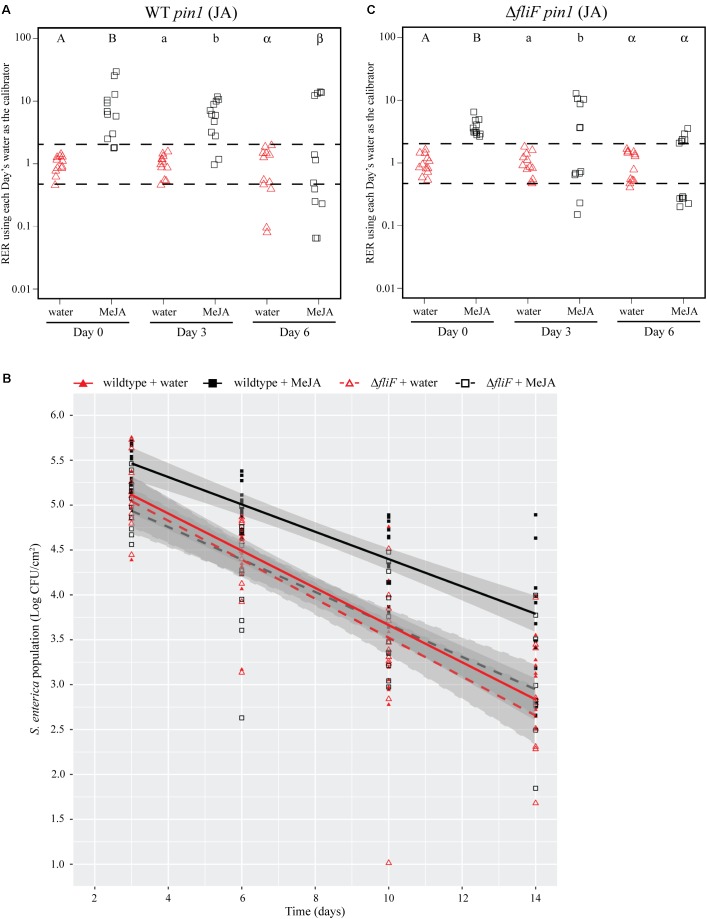
Induction of the JA response with a chemical analog increases *S.* Typhimurium survival over time. **(A,C)** Plant *pin1* gene expression was quantified at days 0, 3, and 6 post-inoculation with wildtype *S.* Typhimurium **(A)** or the *fliF*::Kan mutant **(C)** for plants sprayed with water (red triangles) or methyl jasmonate (MeJA; black squares) and displayed as relative expression ratios (RER) using transcription on plants treated with water as the calibrator. The dashed line indicates a twofold change in transcription, and each symbol represents data collected from one tomato plant. Transcript levels were quantified from three biological replicates with samples taken from four plants per treatment per time point. Letters denote significant differences between treatments within a single time point (*P* < 0.05). **(B)** Bacterial populations on leaves were monitored over time following either treatment with water (red triangles) or MeJA (black squares) 24 h prior to bacterial dip inoculation. All data points from three independent experiments were combined and presented as *S.* Typhimurium populations (log CFU/cm^2^) over a 14-day period. Lines (red, solid: wildtype *S. enterica* after water treatment; black, solid: wildtype *S.* Typhimurium after MeJA treatment; red, dashed: *fliF*::Kan after water treatment; black, dashed: *fliF*::Kan after MeJA treatment) correspond to a linear regression model, and shaded areas correspond to the respective 95% confidence interval.

**FIGURE 5:**
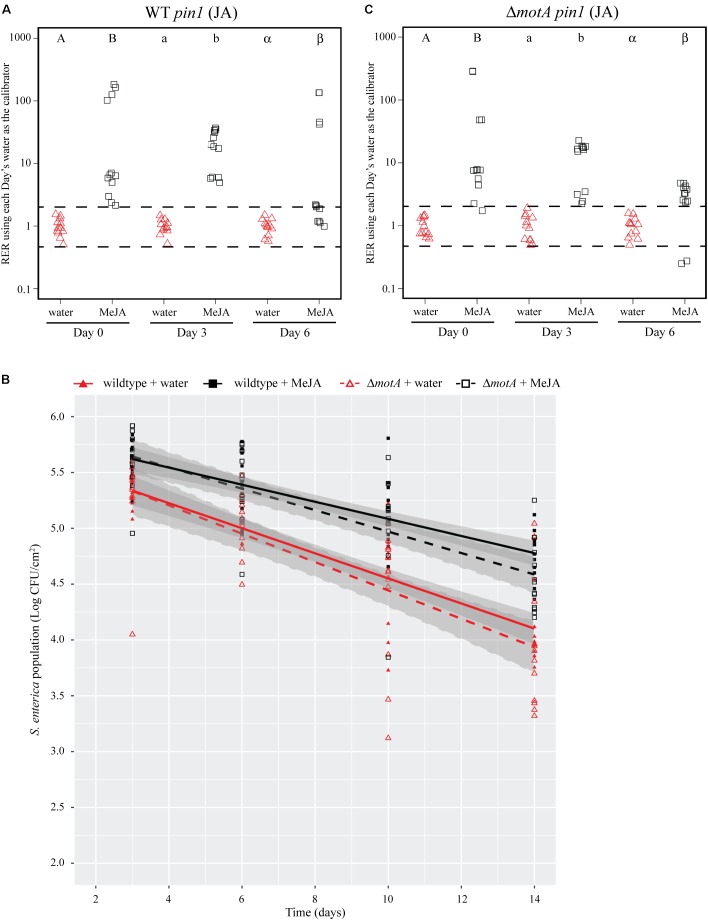
Induction of the JA response with a chemical analog increases *S.* Typhimurium survival over time. **(A,C)** Plant *pin1* gene expression was quantified at days 0, 3, and 6 post-inoculation with wildtype *S.* Typhimurium **(A)** or the *motA*::Kan mutant **(C)** for plants sprayed with water (red triangles) or MeJA (black squares) and displayed as relative expression ratios (RER) using transcription on plants treated with water as the calibrator. The dashed line indicates a twofold change in transcription, and each symbol represents data collected from one tomato plant. Transcript levels were quantified from three biological replicates with samples taken from four plants per treatment per time point. Letters denote significant differences between treatments within a single time point (*P* < 0.05). **(B)** Bacterial populations on leaves were monitored over time following either treatment with water (red triangles) or MeJA (black squares) 24 h prior to bacterial dip inoculation. All data points from three independent experiments were combined and presented as *S.* Typhimurium populations (log CFU/cm^2^) over a 14-day period. Lines (red, solid: wildtype *S.* Typhimurium after water treatment; black, solid: wildtype *S.* Typhimurium after MeJA treatment; red, dashed: *motA*::Kan after water treatment; black, dashed: *motA*::Kan after MeJA treatment) correspond to a linear regression model, and shaded areas correspond to the respective 95% confidence interval.

To differentiate the effects of leafhoppers on the plant immune response from other potentially beneficial impacts of insect infestation on *S.* Typhimurium, we artificially induced the JA defense response with exogenous application of a chemical JA analog, MeJA. Due to biosafety limitations when studying *S.* Typhimurium, MeJA treatment was performed 24 h prior to bacterial dip-inoculation instead of concurrently as done in experiments with leafhoppers (**Figure 1**). Leaves sprayed with MeJA displayed an induction of *pin1* expression that was comparable to the leafhopper-induced JA response (**Figure 3**) and lasted through day 6 post-bacterial inoculation (**Figures 4A**, **5A**). MeJA treatment led to a significant increase in *S.* Typhimurium population persistence compared to negative control plants that were sprayed with sterile water (**Figure 4B**).

### The Bacterial Flagellum Is Necessary for Enhanced *S.* Typhimurium Persistence After MeJA Treatment

**FIGURE 6:**
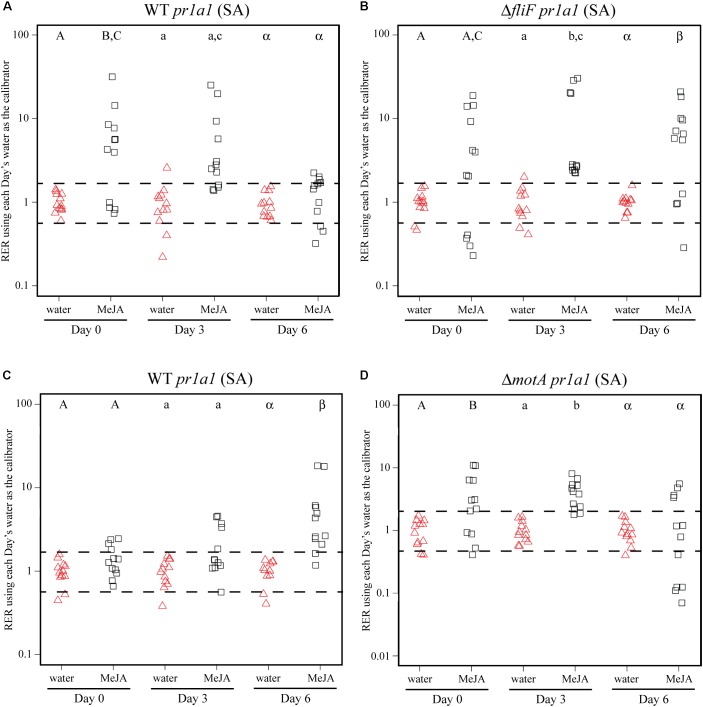
MeJA treatment and *S.* Typhimurium inoculation lead to activation of the SA response. Plant *pr1a1* gene expression was quantified at days 0, 3, and 6 post-inoculation with wildtype *S.* Typhimurium (WT; **A,C**), the *fliF*::Kan mutant **(B)** or the *motA*::Kan mutant **(D)** for plants sprayed with water (red triangles) or MeJA (black squares) and displayed as relative expression ratios (RER) using transcription on plants treated with water as the calibrator. The *fliF*::Kan and *motA*::Kan mutants were tested in separate experiments, and the corresponding wildtype data are displayed for each experiment. The dashed line indicates a twofold change in transcription, and each symbol represents data collected from one tomato plant. Transcript levels were quantified from three biological replicates with samples taken from four plants per treatment per time point. Letters denote significant differences between treatments within a single time point in each experiment (*P* < 0.05).

To identify bacterial factors that impact the connection between *S.* Typhimurium persistence and the immune response, we examined bacterial populations in two motility mutants: *fliF*::Kan and *motA*::Kan. As motility has previously been linked to bacterial persistence on plant leaves (Haefele and Lindow, 1987; Lindow et al., 1993) and the *S. enterica* flagellum is a known stimulus of the plant immune response (Garcia et al., 2014), we hypothesized that the flagellum may play a role in bacterial persistence in our experimental system. The *fliF* gene encodes the inner membrane ring required for construction of the bacterial flagellum, and *motA* encodes one of the membrane proteins that acts as a stator in the flagellar motor (Block and Berg, 1984; Homma et al., 1987a,b; Blair and Berg, 1990). Although both mutants are non-motile, the *fliF*::Kan mutant does not make a flagellum while the *motA*::Kan mutant makes a non-functional flagellum (Block and Berg, 1984; Homma et al., 1987a,b; Blair and Berg, 1990). Plants were treated with MeJA (as described above) and then dip-inoculated with either wildtype, the *fliF*::Kan mutant, or the *motA*::Kan mutant. While MeJA treatment resulted in a significant increase in the persistence of wildtype *S.* Typhimurium, populations of the *fliF*::Kan mutant were not significantly different on plants treated with MeJA or water (**Figure 4B**). Examination of *pin1* expression revealed that JA induction did not persist as long in plants inoculated with the *fliF*::Kan mutant compared to wildtype (**Figures 4A,C**). Unlike the *fliF*::Kan mutant, MeJA enhanced persistence of the *motA*::Kan mutant compared to the water control (**Figure 5B**), and JA induction continued through day 6 after inoculation for the *motA*::Kan mutant (**Figure 5C**). To further characterize the effects of these treatments on plant immunity, we also monitored transcription of the SA response using *pr1a1* primers (**Figure 6**). Expression of *pr1a1* was significantly induced after MeJA treatment and bacterial dip-inoculation in all tested strains at variable time points (**Figure 6**).

## Discussion

Although best characterized for its role as a human enteric pathogen, *S. enterica* colonizes plants as part of its lifecycle and as a means for returning to an animal host (for review, see Barak and Schroeder, 2012). Although *S. enterica* cannot disrupt plant cells such as plant pathogens to release nutrients from the leaf surface, the bacteria successfully survive in the plant environment and persist for months with a slow decline in populations over time (Islam et al., 2004a,b). Biomultipliers, such as phytobacterial pathogens or phytophagous insects, enhance *S. enterica* survival by altering the plant environment to the benefit of the enteric pathogen (Kwan et al., 2013; Soto-Arias et al., 2013; Potnis et al., 2014, 2015). Understanding the mechanisms for the interaction between *S. enterica* and these biomultipliers is important in order to reduce the risk of human disease from contaminated produce. In this study, we describe how phytophagous insects modulate plant immunity, and further, we provide a mechanism linking the bacterial flagellum and the plant JA response to *S. enterica* persistence.

Here, we examined the impact of one common phytophagous insect, the Aster leafhopper, and further characterized its role as a biomultiplier of *S.* Typhimurium populations. Using a similar methodology as described in this study (**Figure 1**), we had previously shown that leafhoppers enhance *S. enterica* persistence on lettuce plants (Soto-Arias et al., 2013). Those experiments demonstrated that, regardless of treatment, *S. enterica* populations slowly decline over time. Infestation with leafhoppers increased the longevity of a cocktail of *S. enterica* strains (Cubana, Enteritidis, Newport, Poona, Schwarzengrund, Baildon, and Mbandaka) on lettuce leaves compared to plants without insect infestation (Soto-Arias et al., 2013). In this study, we demonstrate that, in the absence of leafhoppers, *S.* Typhimurium populations on tomato leaves also show a slow decline (**Figure 2**). However, two noticeable differences can be observed when comparing the data from lettuce to those collected from tomato. First, in the absence of leafhoppers, the decline in *S.* Typhimurium populations on tomato leaves appears to be slower (**Figure 2**) than the decline that was observed on lettuce (Soto-Arias et al., 2013). Second, in contrast to the experiments performed with lettuce (Soto-Arias et al., 2013), leafhopper infestation on tomato plants led to a constant *S.* Typhimurium population with no appreciable loss of overall numbers over the course of the experiment (**Figure 2**). The maintenance of a steady bacterial population level indicates that either the bacteria remain viable with no significant growth, or bacterial replication rates are equivalent to bacterial death, leading to no observable changes in overall bacterial numbers. The differences observed between bacterial populations on these two plants could also reflect differences in leafhopper feeding behavior on the two plant hosts. Aster leafhoppers overwinter on grains but will infest vegetable crops such as carrot, celery, potato, radish, and lettuce during the growing season (Hagel et al., 1973; Beanland et al., 2005). Although the leafhoppers utilize many different crops as sources of food or refuge, leafhopper reproduction consistently occurs only on lettuce, suggesting lettuce is a more preferred plant host. Thus, on lettuce, the leafhoppers may locate feeding sites quickly and create fewer damage sites (e.g. salivary sheaths) (Miles, 1972; Backus, 1988). Contrastingly, tomato plants, a Solanaceae plant like potato, could be considered a non-preferred host plant for the insects, and the leafhoppers may spend more time probing the plant in search of nutrients. The increased probing has the potential to increase the defense response. Although we do not have data from lettuce as a comparison, we observed a robust induction of the JA response (12- to 236-fold) after 3 days of leafhopper infestation (**Figure 3A**). Interestingly, by 6 days post-infestation, *pin1* levels were not significantly different between water-treated plants and leafhopper-treated plants (**Figure 3C**). This reduction in the JA response over time may suggest that leafhoppers stop feeding at later points in the experiment and may be further evidence that tomato are not a preferred food source. Future experiments could address these questions by examining the lettuce defense responses in comparison to the tomato response presented here and monitoring leafhopper feeding patterns using these plant systems.

We hypothesize that leafhopper-induced alterations to plant immunity provide a benefit to *S.* Typhimurium and extend bacterial survival on the leaf surface. By day 3 post-inoculation, co-inoculation of leafhoppers with *S.* Typhimurium led to a significant induction of the JA response while there was no change in the SA response until day 6 (**Figure 3**). Due to the strong, sustained induction of *pin1* expression, we chose to focus on the role of the JA response in enhancing *S.* Typhimurium persistence. To determine if changes in the JA response are sufficient for the effects on bacterial persistence, plants were treated with the analog MeJA to activate the immune response in the absence of leafhoppers. If MeJA treatment provided the same advantage as leafhopper infestation for *S.* Typhimurium populations, then we could rule out other mechanisms that explain the effect that leafhoppers have on bacterial populations, such as providing increased nutrients during insect feeding. Our results demonstrate that MeJA treatment enhanced bacterial persistence (**Figure 4B**), indicating that induction of the JA response is involved in the effect on bacterial populations. Contrasting to the leafhopper experiments (**Figure 2**), *S.* Typhimurium populations continued to decline over time after MeJA treatment (**Figure 4B**). As discussed above, leafhopper infestation resulted in a flat slope for the linear regression line describing bacterial populations while mock-treated plants had slowly declining levels of *S.* Typhimurium (**Figure 2**). In the MeJA experiments, we observed that both the water-treated and the MeJA-treated plants had a sharper decline in *S.* Typhimurium populations compared to the leafhopper experiment (**Figures 4**, **5** compared to **Figure 2**). Statistical analyses indicate that we cannot attribute the differences in slopes to the different experimental procedures used for these experiments. In the MeJA experiments, plants were sprayed with water or MeJA 24 h prior to bacterial inoculation while this treatment was not used in the leafhopper experiment. We found that bacterial populations on water-treated plants from the leafhopper experiment (**Figure 2**) were not significantly different from populations on water-treated plants in the MeJA *motA*::Kan experiment (**Figure 5**) at days 3, 6, and 7 but both populations were statistically different than those seen on water-treated plants in the MeJA *fliF*::Kan experiment (**Figure 4**) on those days (*P* < 0.05). The leafhopper experiment data (**Figure 2**) were statistically different from both MeJA experiments (**Figures 4**, **5**) by day 10 (*P* < 0.05). Biological variability does not explain these results as biological replicates within each experiment (each figure represents three independent experiments for those treatments) were not significantly different from one another (*P* > 0.05). This statistical analysis does preclude any conclusions that can be made about the sufficiency of JA induction on bacterial population enhancement. However, whole genome transcriptome analyses examining the plant response to insect herbivory demonstrate that plants alter transcription in multiple pathways, including primary metabolism, signaling, cell wall modification, and oxidative stress (Schwachtje and Baldwin, 2008; Kerchev et al., 2012). In the face of such a widespread response, we would have been surprised to find that JA induction alone was sufficient to effect bacterial persistence. Thus, although induction of the JA response (by leafhopper infestation or MeJA treatment) provides a benefit to *S.* Typhimurium, we hypothesize that additional mechanisms are likely present that also affect bacterial survival. When leafhoppers feed on leaves, they must excrete excess carbohydrates to counterbalance the difference in osmolarity that they encounter in the phloem (Wilkinson et al., 1997). These excretions are termed “honeydew,” and we have data showing that *S. enterica* can use honeydew as a nutrient source (J. Dundore-Arias, personal communication). Release of nutrients from the leaf during leafhopper feeding or excretion of honeydew by the insects could provide a nutrient source for *S. enterica* in the otherwise nutrient poor environment on the leaf surface. Utilization of insect byproducts has been described for other human enteric pathogens as regurgitation by house flies provides nutrients for pathogenic *Escherichia coli* and leads to bacterial replication on spinach leaves (Wasala et al., 2013). Future analysis of honeydew as a nutrient source for *S. enterica* and quantification of leafhopper excretions on the leaf surface will elucidate the impact of nutrient availability on *S. enterica* survival.

The data presented in this study demonstrate that the presence of leafhoppers alter hormone production (**Figure 3**). Alone, *S.* Typhimurium induces the SA response; tomato plants that are inoculated with *S.* Typhimurium and infested with leafhoppers activate both the JA and SA pathways. Similarly, MeJA induction of the JA response in conjunction with *S.* Typhimurium inoculation leads to activation of both the JA and SA pathways (**Figures 4–6**). In many cases, activation of one pathway suppressors activation of the other pathway (Pena-Cortes et al., 1993; van Wees et al., 2000; Spoel, 2003; Van der Does et al., 2013; Wei et al., 2014). In nature, plants are often invaded by multiple pests at the same time. The antagonism between the SA and JA pathways allows the plant to prioritize one pathway over the other to provide the most effective response to its attackers (Kunkel and Brooks, 2002; Beckers and Spoel, 2006; Koornneef and Pieterse, 2008). Despite much work describing the antagonistic nature of the SA and JA pathways, it is now accepted that the story is more complicated than the simple model where one pathway is activated while the other pathway is inhibited. For example, evidence from *A. thaliana* shows that treatment with low concentrations of the two hormones transcriptionally activates both defense pathways while higher concentrations produce the more typical antagonistic effect (Mur et al., 2006). Thus, the relationship between the SA and JA defense responses is dependent on the relative concentration of each hormone and also may be specific to the organism that initially stimulates the response. The data from this study suggest that simultaneous introduction of a phytophagous insect and a bacterium leads to a synergy between the SA and JA pathways. One limitation of this work is the localized nature of the sampling technique. In the leafhopper experiment (**Figures 2**, **3**), leaf samples for bacterial population and RNA analyses were taken from the localized insect infestation site within clip cages. Although the plant immune response initially responds in a localized manner, the plant prepares for further pathogen invasion in a systemic fashion (for review, see Henry et al., 2013). Immune activation leads to induction of the systemic acquired resistance in distal leaves and often includes accumulation of plant hormones like SA. To expand on our understanding, future studies examining the systemic response to these experimental conditions could reveal more information regarding the potential synergistic effects of the SA and JA response in this system.

To further define the mechanisms that enhance bacterial survival, we examined the importance of the flagellum, with known roles in motility and immune recognition, in bacterial persistence on tomato leaves. For these experiments, we characterized two mutants with defects in structural or functional aspects of flagellar-based motility. The non-flagellated *fliF*::Kan mutant is not motile and does not secrete the immunostimulatory flagellin subunit while the *motA*::Kan mutant produces an intact flagellum but is not motile due to a non-functional flagellar motor (Block and Berg, 1984; Homma et al., 1987a,b; Blair and Berg, 1990). Although non-motile, the *motA*::Kan mutant could use the non-functional flagellum for attachment, or the presence of extracellular flagellin monomers in the mutant strain could stimulate an immune response. The difference in the two mutant strains provides a distinction between a requirement for motility (*fliF*::Kan) and a requirement for the presence of the flagellum for attachment or stimulation of an immune response (*motA*::Kan). After pretreatment with MeJA to induce the JA response, the *motA*::Kan mutant displayed an increase in bacterial persistence (**Figure 5B**) while the *fliF*::Kan mutant populations were not significantly different on plants treated with MeJA or water (**Figure 4B**). The *motA*::Kan results indicate that motility is not required for *S.* Typhimurium to benefit from the MeJA-induced JA response as both the wildtype and non-motile *motA*::Kan strain have enhanced persistence after MeJA treatment. The *fliF*::Kan data suggest that the presence of the flagellum is needed to enhance bacterial persistence, perhaps through a mechanism involving attachment or recognition by the immune system. We predict that a connection to the immune response is more likely due to evidence that *S.* Typhimurium can attach to plant leaves in a flagellum-independent manner (Berger et al., 2009). Previous work demonstrated that, although a *S. enterica* serovar Senftenberg flagellum mutant has reduced attachment to basil leaves, *S.* Typhimurium (the serovar used in this study) can attach to leaves even in the absence of the flagellum (Berger et al., 2009). Thus, we hypothesize that the effects on immune recognition are a more likely mechanism to explain the role of flagella in this system.

**FIGURE 7:**
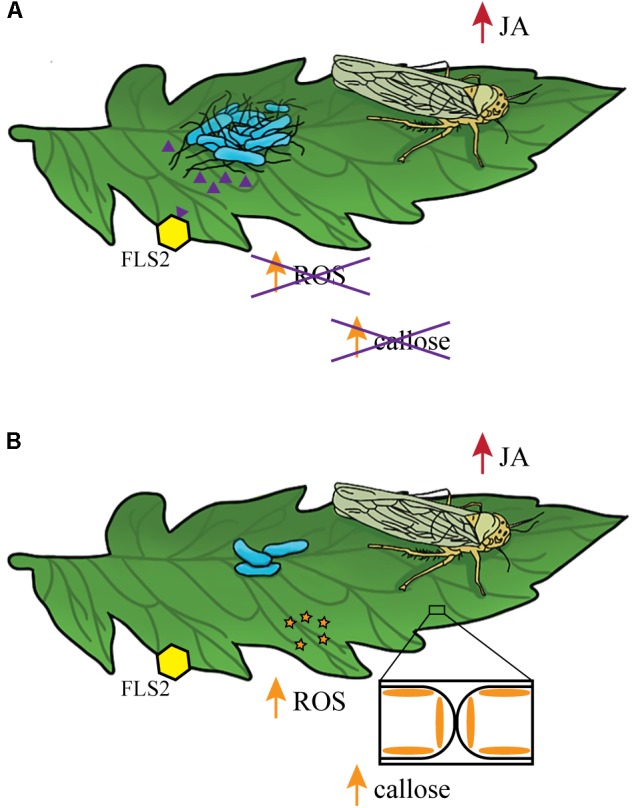
Model describing the link between the bacterial flagellum and the positive effects of leafhopper infestation on *S.* Typhimurium persistence. **(A)** Leafhopper infestation leads to increased populations of flagellated *S.* Typhimurium (blue) on the leaf surface. Leafhopper-induction of the JA response (red) in combination with recognition of extracellular *S.* Typhimurium flg22 peptides (purple triangles) by FLS2 (yellow hexagon) inhibit ROS activation and callose deposition. **(B)** Although leafhoppers induce the JA response (red), non-flagellated *S.* Typhimurium populations (blue) decline due to activation of ROS activation (orange stars) and callose deposition.

The best characterized bacterial PAMP is the flagellin peptide flg22. This conserved N-terminal region of the flagellin protein is present in both *S.* Typhimurium flagellin proteins FliC and FljB (Garcia et al., 2014) and is recognized by the PRR flagellin-sensing protein 2 (FLS2). FLS2 recognition activates a cascade of responses categorized as pathogen-triggered immunity (PTI) (for review, see Melotto et al., 2014). A second PRR FLS3 was recently identified in tomato that recognizes a distinct region of flagellin called flgII-28 (Hind et al., 2016). Previous work in *A. thaliana* demonstrated that activation of the JA and SA pathways can influence PTI (Yi et al., 2014). Treatment with the flg22 peptide led to SA accumulation, increased ROS production, and increased callose deposition (Meng et al., 2013; Garcia et al., 2014). However, pretreatment with hormone analogs such as the JA mimics coronatine (COR) and MeJA led to a reduction in flg22-induced activation of ROS and callose accumulation (Yi et al., 2014). These results indicate that when the JA response is activated, flg22-induced responses are reduced. However, pretreatment with hormone analogs in the absence of flg22 has no effect on ROS production or callose deposition (Yi et al., 2014). The interaction between the JA response and flg22-induced changes in immunity could explain why wildtype *S.* Typhimurium and the *motA*::Kan mutant benefit from MeJA induction of the JA pathway while the *fliF*::Kan mutant does not (for model, see **Figure 7**). We chose not to include the SA response in our model because, although it is also activated under these conditions, there were no significant differences in *pr1a1* activation between wildtype and the *fliF*::Kan mutant (**Figure 6**), indicating that the SA pathway is not likely to be responsible for the population differences observed for these strains. When inoculated alone, *S. enterica* flg22 stimulates the immune response which rapidly produces increased levels of ROS and callose deposition (Garcia et al., 2014). Pretreatment with MeJA or infestation with leafhoppers in conjunction with *S.* Typhimurium inoculation activates the JA response (**Figures 3**–**5**). Thus, we predict that inoculation with wildtype or the *motA* mutant, which both produce the flg22 peptide, would lead to a reduction in ROS levels and callose deposition (**Figure 7**). Inoculation with the *fliF* mutant, which does not make extracellular flagellin, would result in normal levels of ROS activation and callose deposition in response to immune activation (**Figure 7**). These differential immune responses could explain the differences in bacterial persistence that were observed in the MeJA experiments between the wildtype, *motA*::Kan mutant, and *fliF*::Kan mutant (**Figures 4**, **5**). Without flagella, the *fliF*::Kan mutant must still contend with ROS activation and callose deposition, and induction of the JA pathway is irrelevant to bacterial persistence in the absence of the flagellin protein. Future experiments looking at these downstream outputs of JA- and flg22-induced activation of the immune response could further elucidate the mechanism that leads to enhancement of *S. enterica* persistence.

In summary, this work provides several important contributions to the study of human enteric pathogens in plant hosts. We present another example of a synergistic response between the SA and JA immune pathways that adds to a growing body of work that challenges the dogma of a strictly antagonistic interaction between these two pathways. Further, we define a potential mechanism by which phytophagous insects enhance human enteric pathogen survival on leaves (**Figure 7**). Our data suggest that leafhopper-induced activation of the JA response in conjunction with plant responses to flg22 benefit *S. enterica* survival while also providing evidence that suggests additional mechanisms may remain to be discovered. We have advanced the fundamental knowledge describing how the phyllosphere community is influenced by insect herbivory and filled in gaps in understanding the biology of tri-tropic interactions between an enteric human pathogen, a phytophagous insect, and a plant host.

## Author Contributions

KC, RG, and JB conceived the study, analyzed the data, and wrote the manuscript. KC conducted the assays. All authors have read and approved the final manuscript.

## Conflict of Interest Statement

The authors declare that the research was conducted in the absence of any commercial or financial relationships that could be construed as a potential conflict of interest.

## Footnotes

1 http://www.R-project.org
